# Outcomes after ventral mesh removal: a multicentric cohort study

**DOI:** 10.1007/s10029-026-03682-y

**Published:** 2026-04-28

**Authors:** Julie Turmine, Gaëtan-Romain Joliat, David Moszkowicz, Elena Belloni, Benoît Romain, Guillaume Passot

**Affiliations:** 1 Department of General Surgery and Surgical Oncology, Centre Hospitalier Universitaire Lyon Sud, 165, chemin du Grand Revoyet, Pierre-Bénite, 69495 France; 2 Department of Visceral Surgery, Lausanne University Hospital CHUV, University of Lausanne (UNIL), Lausanne, Switzerland; 3 Department of General and Digestive Surgery, Université Paris Cité, Hôpital Louis-Mourier, DMU ESPRIT - GHU AP-HP Nord - Université Paris Cité, Colombes, France; 4 Department of General and Digestive Surgery, Hautepierre Hospital, Strasbourg, France

**Keywords:** Infection, Intraoperative complications, Postoperative morbidity

## Abstract

**Purpose:**

In case of infection, recurrence, chronic pain or acute bowel obstruction, removal of a ventral mesh might be necessary. While infrequent, ventral mesh removal can be associated with important postoperative morbidity. The aim of this study was to assess if postoperative major complications occurred more frequently after removal of an intraperitoneal mesh compared to a retromuscular mesh.

**Methods:**

A retrospective cohort study was performed. Patients from 3 centers in France were included. Patients were included if they underwent removal of a ventral mesh for any reason and if older than 18. Patients with intraperitoneal and retromuscular meshes were compared. Overall and major (≥grade 3a) complications were defined according to the Clavien-Dindo classification. The primary endpoint was the rate of major complications.

**Results:**

A total of 101 patients were included (50 women, 50%; median age 63, IQR 53–72; median body mass index 29, IQR 27–32; 20 active smokers, 20%; 35 diabetic patients, 35%). Intraperitoneal mesh was present in 74 patients (73%), retromuscular mesh in 22 patients (22%), and subcutaneous mesh in 5 patients (5%). Postoperative complications occurred in 40 patients in the intraperitoneal group (54%) and in 12 patients in the retromuscular group (55%, *p* = 0.967). Major complication rate was higher in the intraperitoneal group compared to the retromuscular group (18/74 = 24% vs. 1/22 = 5%, *p* = 0.041). On multivariable analysis, age, smoking, and intraperitoneal mesh removal were found as independent predictors of major complication.

**Conclusion:**

In this multicentric cohort of patients, removal of intraperitoneal mesh compared to removal of retromuscular mesh was associated with a higher risk of major postoperative morbidity. Use of intraperitoneal mesh for ventral hernia should therefore be cautiously and restrictively considered.

**Supplementary Information:**

The online version contains supplementary material available at 10.1007/s10029-026-03682-y.

## Introduction

Abdominal wall surgery is very common worldwide and nowadays mesh repair is largely recommended for primary and incisional ventral hernia repairs to decrease the risk of recurrence [1, 2]. If possible, mesh should be primarily positioned in the retromuscular or preperitoneal plan [3, 4]. However, intraperitoneal mesh position has been initially largely used since the early 1990’s, due to its relative low surgical complexity, rapid operation duration, short-term learning curve and is still routinely and often used. Recently, this technique has been criticized for its short- and long-term risks of life-threatening complications compared to extraperitoneal techniques but scientific evidence remains scattered, mainly due to the short follow-ups of published studies [5, 6].

Even if recurrence rates have decreased after ventral hernia repair, other complications such as infections, enterocutaneous fistulas, bowel obstructions or adhesions rendering a subsequent abdominal surgery more complex have been reported [6]. The non negligeable postoperative morbidity imposes to question and objectively evaluate our surgical techniques [7–9]. The number of mesh explantation for various reasons (infection, pain, intestinal obstruction, recurrence) has also increased, leading to new surgical challenges during abdominal wall repair [10].

The aim of the present study was to assess the rates of postoperative complications (major and overall complications) after removal of an intraperitoneal mesh compared to removal of an extraperitoneal mesh.

## Methods

### Patients and database

A multicentric retrospective study was performed. Consecutive patients who underwent total ventral mesh removal in 3 French hernia surgery expert centers between 2018 and 2024 were considered. The surgery for mesh removal was performed at least one year after the primary surgery for mesh placement.

Patients were included if they underwent total ventral mesh removal without restriction on etiologies (infection, recurrence, pain, or obstruction) and if older > 18 years. Patients with inguinal mesh removal were excluded. The specific type of mesh and the mesh characteristics (such as coating) were not available at time of surgery because most patients were referred from other centers, but all intraperitoneal meshes where coated meshes.

Data were extracted in each center from medical records and then centralized anonymously in one database. Postoperative complications were defined based on the Dindo-Clavien classification [11]. Major complications were defined as Dindo-Clavien grade ≥3.

The study was conducted in accordance with the Helsinky decalaration, and received Internal Review Board approval (No. Internal Review Board Hospices Civils de Lyon IRB 00013204; study No: 22_446).

### Endpoints, management, and definitions

The primary endpoint was the rate of major complications after mesh removal. Secondary endpoints were the overall morbidity rate, operation duration, intraoperative bowel injury, length of stay, chronic pain, and recurrence rate.

Preoperative botulinum toxin injection was used if hernia defect was > 10 cm or in case of large concomitant median and lateral hernias. Preoperative progressive pneumoperitoneum was used in case of loss of domain. Loss of domain was defined using the Sabbagh method [12].

Mesh positions were defined as intraperitoneal, retromuscular, or subcutaneous (onlay).

Pain was considered as chronic if the duration lasted > 6 months postoperatively.

### Statistical analysis

Continuous data were summarized using median and interquartile range, while categorical data were summarized with number and percentage. Comparisons between continuous variables were made with Mann Whitney U tests and between categorical variables with chi-square test. The onlay group was not included in the comparative analysis as the population was limited (*n* = 5). To find risk factors of major complications, a binary multivariable logistic regression was performed. Only variables with *p* < 1% on univariable analysis were included.

A p-value < 5% was considered statistically significant. SPSS 29.0 for Mac OS X was used for all statistical analyses (IBM, Chicago, Illinois, USA).

## Results

A total of 101 patients were included in the cohort (Paris *n* = 50, Lyon *n* = 40, Strasbourg *n* = 11). Fifty patients were women (50%), and median age was 63 (IQR 53–72). Most patients had an ASA score of 2 (*n* = 57, 56%), while 14 (14%) and 30 patients (30%) had an ASA score of 1 and 3, respectively. Median BMI of the cohort was 29.2 kg/m^2^ (IQR 26.7–32.3). Twenty patients were active smokers at time of surgery (20%), and 35 patients were diabetic (35%). Meshes that were removed were positioned intraperitoneally in 74 patients (73%), in the retromuscular plane in 22 patients (22%), and subcutaneously in 5 patients (5%). One patient was operated in an emergency setting due to bowel obstruction, and 2 presented an entero-atmospheric fistulas who were operated on within a couple of days after urgent admission.

## Comparison between patients with intraperitoneal and retromuscular mesh removal

Characteristics and intraoperative details of patients who had removal of intraperitoneal mesh compared to those who had removal of retromuscular mesh are depicted in Table 1. No differences were found between both groups. A total of 33 patients (45%) and 7 patients (32%) had 2 or more previous repairs of the recurring hernia in the intraperitoneal and retromuscular groups, respectively (*p* = 0.286).

**Table 1:** Characteristics and intraoperative details of patients (*n* = 96) based on the location of the mesh that was removed (intraperitoneal and retromuscular positions)

	Intraperitoneal mesh *N* = 74	Retromuscular mesh *N* = 22	*P*-values
Age, years	63 (55–71)	66 (48–76)	0.827
Women/men	37 (50%)/37 (50%)	8 (22%)/14 (78%)	0.260
Body mass index, kg/m^2^	29 (27–34)	30 (26–31)	0.688
American Society of Anesthesiologists score I/II/III	9 (12%)/42 (57%)/23 (31%)	4 (18%)/11 (50%)/7 (32%)	0.742
Diabetes	24 (32%)	9 (41%)	0.462
Smokers	15 (20%)	4 (18%)	0.829
Immunosuppression	8 (11%)	4 (18%)	0.359
COPD	16 (22%)	3 (14%)	0.409
Botulinum toxin injection	13 (18%)	1 (5%)	0.129
Preoperative pneumoperitoneum	3 (4%)	0	0.307
Cause for reoperation Infected mesh Recurrence* Acute bowel obstruction	45 (61%) 46 (62%) 2 (3%)	12 (55%) 11 (50%) 0	0.815
Absence of hernia	28(38%)	11 (50%)	0.308
Hernia location Median Lateral Parastomal	36 (49%) 9 (12%) 1 (1%)	8 (36%) 2 (9%) 1 (5%)	0.534
Hernia defect size, cm	9 (5–13)	6 (5–9)	0.101
Operative duration, min	180 (120–220)	137 (100–167)	0.177
Laparotomy Robot	74 (100%) 0	21 (95%) 1 (5%)	0.065
Intraoperative bowel injury	13 (18%)	3 (14%)	0.664
Intraoperative bowel resection	6 (8%)	1 (4%)	0.572
Modified VHWG classification I/II/III	5 (7%)/18 (24%)/51 (69%)	0/7 (32%)/15 (68%)	0.797
Position of the new mesh Intraperitoneal Retromuscular/preperitoneal Onlay	11 (15%) 21 (28%) 11 (15%)	2 (8%) 6 (24%) 3 (12%)	0.875
Component separation Anterior Posterior	2 (3%) 12 (16%)	0 3 (12%)	0.385

* 30 and 5 patients had concomitant mesh infection and hernia recurrence in the intraperitoneal group and in the retromuscular group

*COPD* chronic obstructive pulmonary disease, *VHWG* ventral hernia working group

Morbidity rates (overall complication rates) were similar between patients with intraperitoneal mesh removal and those with retromuscular mesh removal (40/74 vs. 12/22, *p* = 0.967). However, major complications occurred more frequently after intraperitoneal mesh removal than retromuscular mesh removal (18/74 vs. 1/22, *p* = 0.041). In the retromuscular group, the major complication was a deep wound infection, that did not require redo surgery or radiological drainage. Regarding the major complications presented in the intraperitoneal group, 2 patients developed a multiorgan failure leading to 1 death; 16 patients were reoperated and 2 required radiological drainage. Regarding the type of complications, 9 patients presented general complications (as bowel ileus (2 patients), pneumonia (2 patients), anemia (2 patients), cardiovascular issues (2 patients), MOF (2 patients), and 2 presented more than one general complications), and 16 presented with surgical site occurrence (12 associated with the wound, 3 intra-abdominal infection, and 1 entero-atmospheric fistula).

The mean follow-up was 14 months (IC 9%: 10–19). No other differences, including recurrence rates, were found between both groups. Postoperative outcomes are summarized in Table 2.

**Table 2:** Postoperative outcomes of patients (*n* = 96) based on the location of the mesh that was removed (intraperitoneal and retromuscular positions)

	Intraperitoneal mesh *N* = 74	Retromuscular mesh *N* = 22	*P*-values
Postoperative ICU stay	11 (15%)	0	11 (15%)
Morbidity			0.967
Minor	22 (30%)	11 (50%)	0.079
Major	18 (24%)	1 (5%)	**0.041**
SSO	34 (46%)	10 (45%)	0.968
LoS, days	6 (4–10)	6 (2–8)	0.695
Percutaneous drainage	2 (3%)	0	0.436
Chronic pain	4 (5%)	1 (5%)	0.873
Recurrence	17 (23%)	4 (18%)	0.633

*ICU* intensive care unit, *SSO* surgical site occurrence, *LoS* length of stay

Significant p-values are in bold

### Risk factors of major complications

In patients who underwent removal of intraperitoneal or retromuscular mesh (*n* = 96), age (OR 1.1, 95% CI 1.0–1.2.0.2, *p* = 0.010), active smoking (OR 4.5, 95% CI 1.2–16.2, *p* = 0.023), and intraperitoneal mesh location (OR 8.9, 95% CI 1.0–76.9.0.9, *p* = 0.047) were found to be independent predictive factors of major complications. Summary of the uni- and multivariable analyses is shown in Table 3.

**Table 3:** Multivariable analysis (binary logistic regression) of predictive factors of major morbidity in patients who underwent intraperitoneal or retromuscular mesh removal (*n* = 96)

	Univariable OR (95% CI)	*P*-values	Multivariable OR (95% CI)	*P*-values
Age, years	1.1 (1.0-1.1)	** 0.021**	1.1 (1.0–1.2.0.2)	0.010
BMI, kg/m^2^	1.1 (1.0–1.2.0.2)	0.157		
Diabetes	1.0 (0.4–3.0.4.0)	1		
Smoker	3.3 (1.1–10.2)	**0.035**	4.5 (1.2–16.2)	**0.023**
Intraperitoneal mesh removal (ref: retromuscular)	7.1 (0.9–55.6)	0.063	8.9 (1.0–76.9.0.9)	**0.047**
Component separation	1.3 (0.4–4.6)	0.767		

*BMI* body mass index

Significant p-values are in bold

### Subgroup of patients with infected mesh

In the subgroup of infected mesh (*n* = 62), median age and BMI were 65 (IQR 53–73) and 29 kg/m^2^ (IQR 25–32), respectively. Twenty-nine patients (47%) were women, 9 patients were active smokers (15%), and 23 patients had diabetes mellitus (37%).

Intraperitoneal meshes were removed in 45 patients (73%), retromuscular meshes in 14 patients (23%), and subcutaneous meshes (onlay) in 3 patients (4%). No difference in overall morbidity was found between patients who underwent intraperitoneal mesh removal compared to those who underwent retromuscular mesh removal (26/45 vs. 10/14, *p* = 0.360). Major morbidity was similar in both groups (14/45 vs. 1/14, *p* = 0.072).

## Discussion

The present article reports the results of a multicentric study including a large cohort of patients who underwent ventral mesh removal. The main finding was that major morbidity was higher after removal of an intraperitoneal mesh compared to a retromuscular mesh (24% vs. 5%, *p* = 0.041). Furthermore, removal of an intraperitoneal mesh was found as an independent predictor of major morbidity. In this multicenter study, the complications reported after intraperitoneal mesh removal were severe: 2 multiorgan failures leading to 1 death, 16 reoperations, and 1 patient developing an entero-atmospheric fistula.

Hayden et al. [13] assessed the rate of subsequent abdominal surgery after a ventral hernia repair in a retrospective study. They did not find any higher surgery complexity due to intraperitoneal mesh but this mesh location induced a higher rate of reoperation (22% of patients with intraperitoneal mesh vs. 14% of patients with extraperitoneal mesh, *p* < 0.001).

Regarding infected meshes, Franchi et al. [7] performed a monocentric study on infected meshes and assessed the possibilities of conservative treatment. The results showed that it might be an option but with high risk of failure (especially in case of chronic infection), letting a wide place for mesh removal in this context. In the present study, no differences in outcomes were found in the subgroup of infected meshes. This could be related to the fact that, in an infected environment, postoperative outcomes are potentially more associated to the septic conditions than to the location of the mesh.

One argument against intraperitoneal mesh use is the occurrence of adhesions with the bowel, inducing a potentially more complex adhesiolysis in case of intra-abdominal access need. While this item was not available in our data, it was found that the rate of enterotomy was similar in both groups. However, the rate of major morbidity was higher in the intraperitoneal group, potentially associated with secondary peritonitis due to bowel lesions, and the operation duration showed a non significant trend of being longer in the intraperitoneal group.

One of the first studies to demonstrate the increased rate of complication due to intraperitoneal mesh was conducted in 2007 by Halm et al. [14] with 77% of postoperative complications. However, this result must be counterbalanced by the fact that those meshes were unprotected polypropylene, which are no longer used in current practice. In 2016, Patel et al. [15] showed that 17% of patients with an history of intraperitoneal mesh ventral hernia repair underwent a subsequent abdominal surgery within a mean of 2.2 years. In this study, they demonstrated that a reintervention with an intraperitoneal mesh was safe, outside of the context of bowel obstruction due to adhesions with the mesh. Years before that, Snyder et al. [16] did not show any differences of inadvertent enterotomy during subsequent abdominal surgery between intra- and extraperitoneal mesh, with a higher rate of 25% of reintervention after ventral incisional hernia repair.

Nowadays, consequences of intraperitoneal placement of a hernia mesh are still largely debated but the present study showed that major complications in case of abdominal reoperation with intraperitoneal mesh removal are higher than with retromuscular mesh. The use of such intraperitoneal mesh should therefore be reserved to complex cases, where the use of extraperitoneal mesh is not feasible. Also, patients and surgeons should be educated and aware of the potential risk of higher surgical complications in case of subsequent abdominal intervention for mesh removal, when using an intraperitoneal mesh.

Several limitations of the study should be acknowledged. The retrospective study design might have induced data collection mistakes, selection bias, and missing data. Regarding the type of meshes and the presence and type of coating, in this study we were not able to determine what type of mesh was removed, and for the intraperitoneal meshes the coating type was also undefined. Moreover, only referral centers were included in the study, which could impact the generalizability of the findings. Another limitation was the fact that the closure strategy was not preliminary defined and could have impacted the operative time. However, the strategy was consistent among centers and applied for all patients irrespective of the mesh location, which limits the risk of difference regarding the groups. The strengths of this study were its multicentric design and the collect of a large cohort of patients who underwent ventral mesh removal.

In conclusion, the present study showed that reoperation for mesh removal after intraperitoneal mesh placement had more major complications compared to removal of a retromuscular mesh, and that intraperitoneal mesh was an independent risk factor of major morbidity. Such results should bring awareness of the potential long-term risk of intraperitoneal mesh when considering to use it.

## Supplementary Information

Below is the link to the electronic supplementary material.

Supplementary Material 1.

Supplementary Material 2.
