# Supplementary material for: Outcomes after ventral mesh removal: a multicentric cohort study
Source: Hernia. 2026 Apr 28;30(1):183. doi: 10.1007/s10029-026-03682-y (PMC13124923; doi:10.1007/s10029-026-03682-y)
Supplement: Supplementary file 1 — Supplementary Material 1. [file 10029_2026_3682_MOESM1_ESM.doc]

Lyon, March, 30^th^ 2026

Subject: **SUBMISSION OF NEW MANUSCRIPT FOR EVALUATION**

We are enclosing herewith a manuscript entitled “Outcomes after ventral mesh removal: a multicentric cohort study” submitted to “Hernia” for possible evaluation.

The study was conducted in accordance with the Helsinky decalaration, and received Internal Review Board approval (No. Internal Review Board Hospices Civils de Lyon IRB 00013204; study No: 22_446). (state in methods in the manuscript) All patients were informed and could use a waiver any time.

Professor Guillaume Passot
